# A review of tuberculosis at the wildlife-livestock-human interface in Zambia

**DOI:** 10.1186/2049-9957-2-13

**Published:** 2013-07-09

**Authors:** Sydney Malama, John Bwalya Muma, Jacques Godfroid

**Affiliations:** 1 Institute of Economic and Social Research, University of Zambia, PO Box 30900, Lusaka, Zambia; 2 Department of Disease Control, School of Veterinary Medicine, University of Zambia, PO Box 32379, Lusaka, Zambia; 3 Department of Food Safety and Infection Biology, Section Biostatistics and Epidemiology, Norwegian School of Veterinary Science, Ullevalsveien 72, Oslo, Norway

**Keywords:** Bovine tuberculosis, Kafue lechwe, Interface, Zoonotic tuberculosis

## Abstract

Zambia’s estimated incidence of all forms of human tuberculosis (TB) is 707/100,000. High prevalence of bovine tuberculosis (BTB) – infection with *Mycobacterium bovis* – in cattle and the Kafue lechwe antelopes (*Kobus leche Kafuensis*) has been reported in the Kafue basin. Consumption of unpasteurised milk and meat products from infected animals poses a risk of transmitting zoonotic tuberculosis to people living at the human-animal interface. Despite the reported high prevalence of BTB in both livestock and wildlife, information on the proportion of human patients infected with *M. bovis* is unknown in Zambia. This paper reviews the available information in English on human, livestock and wildlife TB in Zambia with the purpose of assessing the burden of animal infections with *M. tuberculosis* complex and its public health implications.

## Multilingual abstracts

Please see Additional file 1 for translations of the abstract into the six official working languages of the United Nations.

## Review

### Introduction

Human tuberculosis (TB), although an ancient disease, has re-emerged with devastating consequences on global public health and is currently one of the most widespread infectious diseases. In addition, it is the leading cause of death due to a single infectious agent among human adults in the world [1]. Tuberculosis is caused by members of the *Mycobacterium tuberculosis* complex (MTC), which includes *Mycobacterium tuberculosis, Mycobacterium bovis, Mycobacterium africanum, Mycobacterium caprae, Mycobacterium microti, Mycobacterium pinnipedii* and *Mycobacterium canettii*[1]. Approximately one third of the world’s population is infected with bacteria belonging to the MTC complex, with Sub-Saharan Africa having the highest annual incidence since the advent of HIV and AIDS [2]. The TB bacilli are non-motile, non-sporulating, weakly Gram-positive acid-fast bacilli (AFB) that appear microscopically as straight or slightly curved rods [3].

The World Health Organization (WHO) estimates that the incidence of all forms of TB in Zambia stands at 707/100,000 [4]. *Mycobacterium tuberculosis* is usually transmitted to a human by inhalation of aerosol droplets containing tubercle bacilli which are expectorated from infected individuals with open pulmonary TB [3].

*Mycobacterium bovis (M. bovis),* the bovine tubercle bacilli, is the cause of bovine tuberculosis (in this paper, it will be referred to as BTB when talking about infection to animals and zoonotic tuberculosis when talking about infection to humans). It has a wide range of host animal species, which includes cattle, goats, bisons, antelopes, humans and non-human primates, and can cause disease in susceptible hosts [5].

**Figure 1:**
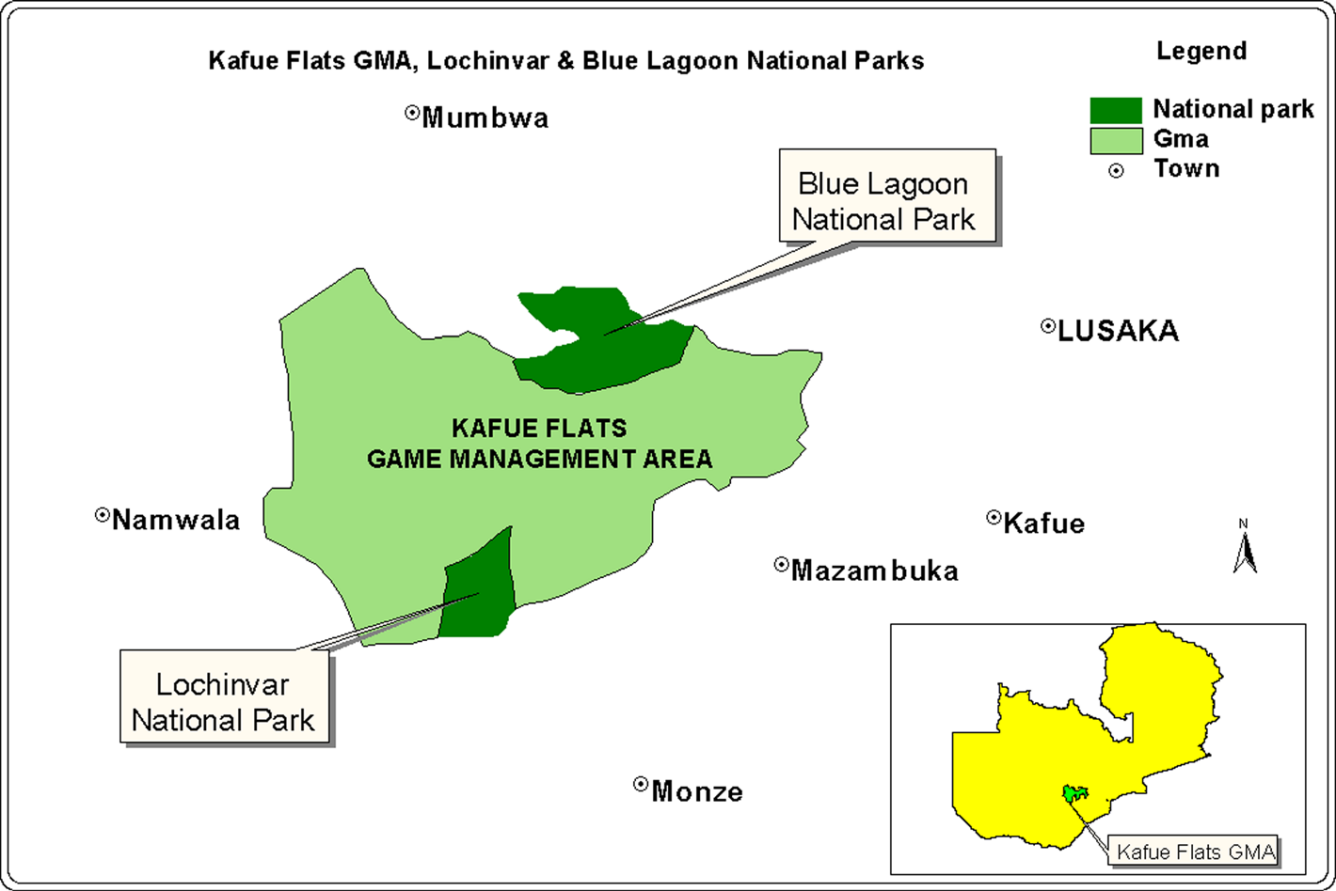
Showing the interface of Kafue basin

High prevalence of BTB in cattle and the Kafue lechwe antelopes (*Kobus leche Kafuensis*) has been reported at the wildlife-livestock interface in the Kafue basin [6,7]. Despite the evidence indicating that cultural and socio-economic factors (among others) increase the likelihood of *M. bovis* transmission between species sharing the same environment (cattle, wildlife and humans), zoonotic tuberculosis remains significantly underrepresented as causal agents of extra pulmonary and pulmonary TB in developing countries, especially in rural regions at the human-animal interface [8]. Humans and animals (both livestock and wildlife) share the same micro-environments and water points within the Kafue basin (see Figure 1), particularly during the dry season, thereby increasing the risk of TB transmission between infected and susceptible hosts [9].

The purpose of this paper is to review the available information on TB in livestock and wildlife in order to identify knowledge gaps, and to assess the burden of animal infections with *M. tuberculosis* and *M. bovis* in addition to their public health importance in Zambia.

### Human demography in Zambia

Zambia is located in south-central Africa and covers an area of about 752,618 square kilometres. The population of Zambia has increased from 7,759,161 in 1990 to 9,885,591 in 2000 and to 13,046,508 in 2010, resulting in an average annual growth rate of 2.8% between 2000 and 2010 [10]. According to the 2010 population figures, 49% of the population is male and 51% is female. The country’s population is characterised by extreme youth with 49.6% of the population being under 15 years of age [10]. The regional population distribution illustrates that 7,978,274 people (61%) reside in rural areas and 5,068,234 (39%) live in urban areas [10]. The rural population in Zambia have based their culture and livelihood around the collection and utilisation of natural resources from the environment [11], which includes activities as diverse as animal husbandry and crop production [12].

### BTB in cattle in the Kafue basin of Zambia

Zambia has approximately three million head of cattle, with an estimated 80% of the national cattle population being held by traditional farmers [13]. The concentration of livestock farming is mainly in three provinces: the Southern, Western and Eastern Provinces. The Kafue Basin area, which is one of the few lacustrine wetlands, supports almost 300,000 cattle [14]. According to Musso et al. (2012), three types of herding systems exist within the Kafue basin. These include village resident herding, with herds kept in the villages; the moving of cattle from villages into the flood plains with regard to the water levels in the plains; and interface herding, where herds are always present within the floodplains and rarely return to the villages, thus being under constant contact with BTB infected wildlife within the floodplains [15].

Bovine tuberculosis (BTB) has been reported to be endemic in the Zambian traditional cattle sector with a high herd prevalence of 49.8% recorded from areas within, and adjacent to, the Kafue basin as far back as 1947 [16,17]. Reports from abattoirs in the Namwala district, located within the Kafue basin, indicate that 16.8% of the cattle slaughtered were infected with BTB based on the presence of typical TB lesions [6].

### BTB in the wildlife in the Kafue basin

The Kafue lechwe (*Kobus leche Kafuensis*) is a medium-sized, semi-aquatic antelope with a population of 44,000 that is endemic to the Kafue flats [18]. The history of BTB in the Kafue lechwe dates as far back as 1954 when it was diagnosed from the Lochinvar National Park, which is located within the Kafue basin [19]. In 1972, Gallagher et al. estimated that BTB was responsible for the deaths of at least 20% of lechwe annually on the southern bank of the Kafue flats [19]. A recent study has shown a magnitude of 27.7% of BTB in the Kafue lechwe [18]. This level of BTB in the Kafue lechwe represents a potential risk of transmission of M. bovis to livestock, wildlife and local communities. A resident population of the African buffalo (*Syncerus caffer*) is present in the Kafue basin. In 2011, a study reported that no BTB positive reactors were found in the comparative intradermal tuberculin test (CIDT), suggesting an absence of M. bovis infection in buffaloes [20]. In addition, this study reported that the buffaloes do not come into direct contact with cattle like the lechwe antelopes do. All the cattle keepers and herdsmen in the Kafue basin reported the same observation that cattle will never go near buffaloes and vice versa; however, lechwe and cattle are often observed grazing together.

### Diagnosis of bovine tuberculosis (BTB) in Zambia

Diagnosis of BTB in cattle and wildlife in Zambia offers numerous challenges and difficulties. The presumptive *ante mortem* diagnosis of TB is made using the CIDT (see Table 1) [7], as recommended by the World Organisation for Animal Health (OIE). This test is usually performed by the veterinary services. However, given that no compensation scheme is in place, farmers are reluctant to slaughter their animals, resulting in this diagnosis test not being routinely implemented in Zambia. Therefore, BTB is diagnosed *post mortem* and is based on the presence of gross lesions compatible with BTB in the lungs and/or associated lymph nodes found during meat inspection in the abattoir. Carcasses are declared fit for human consumption once the organs showing gross lesions are removed according to standard regulations [21]. The laboratory procedures (AFB staining, culture and typing, including molecular methods) (see Table 2) [18,22] are only implemented in the Veterinary Research Institutes and the Faculty of Veterinary Science at the University of Zambia in Lusaka. Furthermore, no resources are allocated for BTB testing and control at the national level as it is not considered a disease of national economic importance.

**Table 1:** Herd prevalence of BTB in cattle determined by cross-section study around the Kafue basin

**Study site**	**Prevalence (%)**	**Method**	**Reference**
Blue lagoon	48	CIDT	Munyeme et al. 2009
Lochinvar	43	CIDT	Munyeme et al. 2009
Kazungula	4	CIDT	Munyeme et al. 2009
Monze	33	CIDT	Cook et al. 1996
Livingstone	1	Necropsy	Anonymous, 1957
Mazabuka	5	Necropsy	Anonymous, 1957
Lusaka	2	Necropsy	Anonymous, 1957
Namwala	17	Necropsy	Anonymous, 1957

**Table 2:** Prevalence of BTB by area based on gross post-mortem examination, Ziehl-Neelsen and culture results from Kafue lechwe tissue samples (n=119) (Munyeme et al. 2010)

**Study area**	**Test method**	**Prevalence (%)**
Lochnivar/Blue lagoon	Necropsy	24.34
Lochnivar/Blue lagoon	Ziehl-Neelsen	17.6
Lochnivar/Blue lagoon	Culture	27.7

Source: Munyeme et al. /Preventive Medicine 95 (2010) 305–308.

### Public health importance of *Mycobacterium tuberculosis* infections in animals

*Mycobacterium tuberculosis* has been incidentally reported from cattle and other livestock animals and may have a negative public health impact. For example, in a study conducted by Ameni et al. on grazing cattle in central Ethiopia, 11.5% of the isolated bacteria, *M. tuberculosis*, was identified [23]. It has also been isolated from livestock and wildlife across the world [1,24-26]. The isolation of *M. tuberculosis* from livestock raises a number of questions relating to the role of livestock as a source of human infections. Of relevance is a study by Srivastava et al. (2008), where *M. tuberculosis* was isolated from milk samples obtained from cattle in India, suggesting that infections may spill back to humans through consumption of unpasteurised milk [27].

### Public health importance of zoonotic tuberculosis in Zambia

In 1998, the WHO reported that 3.1% of tuberculosis cases in humans worldwide are attributable to *M. bovis* and that 0.4-10% of sputum isolates from patients in African countries may be *M. bovis*. This is despite the fact that *M. bovis* is mainly associated with extra pulmonary disease in humans [28]. Data on the prevalence of human disease due to *M. bovis* in Zambia and other developing countries is limited, owing to technical problems posed by identification of this species, such as trained personnel and laboratory facilities [16,29]. Indeed, as a general rule, only Ziehl-Neelsen staining is performed on sputum samples to identify AFB. This technique cannot differentiate between the different species from the genus *Mycobacterium*[30]. However, zoonotic tuberculosis is acquiring increasing recognition in developing countries, including Zambia, as animals and humans share the same environment. This has prompted researchers to evaluate its impact on human health, particularly among pastoral communities. An additional factor that these developing countries are now facing is the HIV/AIDS pandemic, which may favour human-to-human transmission of *M. bovis* leading rapidly to disease [29].

In a more recent study, Gumi et al. (2012) documented, by using molecular tools, an epidemiological link in the zoonotic transmission between livestock and pastoralists of south-east Ethiopia [31]. Zoonotic tuberculosis is an economical and public health threat in developing countries [32]. However, very few studies quantify its economical and public health burden. Recently, a preliminary study suggested that the cost of controlling BTB always exceed the calculated benefits if considered from a purely monetary viewpoint. However, the benefits are likely to outweigh the costs if wider implications of BTB on humans, e.g. avoiding infirmity-related production losses (indirect costs such as time away from farming and on livestock and wildlife) are taken into account [33]. In the Kafue basin, consumption of raw and soured milk is one of the common practices in the local communities. This, therefore, poses a health risk in the event that the milk is drawn from infected animals [34]. Physical contact with cattle and sharing of shelter/space is another common practice in these communities.

Lastly, the Kafue lechwe is hunted for meat, trophies and hides. It is estimated that approximately 80% of lechwe carcasses hunted for meat may be infected with BTB. However, poaching levels specific to lechwe are speculated at 50% of the official annual hunting quota [35]. This places both the poachers and consumers of the meat at risk of contracting zoonotic tuberculosis. Unfortunately, information on zoonotic tuberculosis in humans is virtually absent from the Kafue basin where there is a high prevalence of BTB in livestock and wildlife.

## Conclusion

This review has identified the knowledge gaps in the management of BTB in Zambia. No molecular epidemiological information linking the observed BTB in cattle and wildlife is available. In addition, the review noted that information on the incidences of zoonotic TB is also unavailable despite the reported cultural practices by the people living at the interface. Finally, the review noted that no molecular epidemiological study has been conducted on TB patients living at the interface area and that no information on the presence of *M. tuberculosis* in animals exists.

### The way forward

•There is a need to document epidemiological links of *M. bovis* infections in humans, cattle and Kafue lechwe in the Kafue basin. This could be done by molecular characterisation of isolates obtained from these three species (human, cattle and Kafue lechwe), as well as from milk and the environment [36,37].

•Although *M. tuberculosis* infection has not yet been reported from cattle in Zambia, it has been documented elsewhere [38]. This highlights a need to isolate and differentiate the MTC isolates from cattle to ascertain the presence of *M. tuberculosis*, and to evaluate its epidemiological significance in transmission to humans.

•The interaction of cattle, lechwe and other wildlife, such as buffaloes, at water and grazing points has been documented in the Kafue Basin. Therefore, there is need to conduct BTB field and molecular studies in order to draw epidemiological links between BTB in the different species and to study the risk factors for the transmission of *M. bovis*.

•The fact that buffaloes are not infected with *M. bovis* suggests that environmental contamination is not the preferential route by which wildlife gets contaminated [15]. Moreover, infected lechwe primarily show lesions in the lungs suggesting that infection is acquired by the respiratory route, and the chance of infection increases when lechwe share the same pasture with the infected cattle. Studies on the ecology of *M. bovis* in the Kafue basin are warranted in order to clarify the most important transmission routes at the livestock/wildlife interface.

•Given the high prevalence of BTB in traditional cattle, it is important that surveillance is conducted in communities to ascertain the impact of zoonotic tuberculosis.

## Abbreviations

ABF: Acid-fast bacilli; BTB: Bovine tuberculosis; CIDT: Comparative intradermal tuberculin test; MTC: *Mycobacterium tuberculosis* complex; OIE: World Organisation for Animal Health; TB: Tuberculosis; WHO: World Health Organization.

## Competing interests

The authors declared that they have no competing interests.

## Authors’ contributions

SM contributed to the conception and drafting of the manuscript, JBM and JG contributed to the conception and writing of the manuscript. All authors have read and approved the final manuscript.

## Supplementary Material

Additional file 1 Multilingual abstracts in the six official working languages of the United Nations. Click here for file
