# Supplementary material for: A review of tuberculosis at the wildlife-livestock-human interface in Zambia
Source: Infect Dis Poverty. 2013 Jul 9;2:13. doi: 10.1186/2049-9957-2-13 (PMC3734204; doi:10.1186/2049-9957-2-13)
Supplement: Additional file 1 — Multilingual abstracts in the six official working languages of the United Nations. [file 2049-9957-2-13-S1.pdf]

Translation of the abstract into the six official working languages of the United Nations

مراجعة تداخل حالات السل بين الحياة البرية والمواشي والإنسان في زامبيا

سيدني ملاما، جون بوايلا موما، جاك غَدفرويد

موجز

يقدّر عدد إصابات السل البشري بكافة أشكاله في زامبيا بـ 707 / 100,000. ذكرت التقارير ارتفاع معدل انتشار السل البقري – العدوى بالمتفطرة البقرية- بين المواشي وظباء التيل الأحمر *(Kobus leche Kafuensis)* في حوض نهر كافوي. يشكل استهلاك مشتقات الألبان غير المبسترة ومنتجات اللحوم من المواشي المصابة بالعدوى خطرا لنقل السل الحيواني المصدر إلى الأفراد الذين يعيشون بتماس مع الحيوانات. وعلى الرغم من التقارير التي تشير إلى ارتفاع معدل انتشار السل البقري في كل من الحيوانات البرية والمواشي، فإن المعلومات المتوفرة عن نسبة المرضى من البشر المصابين بعدوى المتفطرة السلية ما تزال غير معروفة في زامبيا. في هذا البحث مراجعة للمعلومات المتوفرة باللغة الانجليزية عن إصابات السل بين الإنسان والمواشي والحيوانات البرية في زامبيا بهدف تقييم العبء الذي تمثله حالات عدوى الحيوانات بالمتفطرة السلية وآثارها على الصحة العامة.

Translated from English version into Arabic by Lina SM, through

TRANSLATORS WITHOUT BORDERS

赞比亚野生动物-家畜-人结核病情况的回顾

Sydney Malama, John Bwalya Muma, Jacques Godfroid

摘要

估计赞比亚人类所有类型的结核病发病率为 707/10 万。据报道在 Kafue 盆地，牛结核病（牛分枝杆菌 *Mycobacterium bovis* 感染）在牛和 Kafue lechwe 羚羊（*Kobus leche Kafuensis*）中的发病率较高。食用受感染动物的未消毒牛奶和肉类产品会引起将人兽共患结核病传播到人类的风险。尽管在家畜和野生动物中牛结核病的发病率较高，但是赞比亚人感染牛分枝杆菌的比例还不清楚。本文对赞比亚人、牲畜和野生动物结核病可用的英文资料进行了回顾，以期评估动物感染结核分枝杆菌复合群（*M. tuberculosis* complex）的疾病负担及其对公众健康的影响。

Translated from English version into Chinese by Yang Pin, through

Étude de la tuberculose à l'interface faune-bétail-homme en Zambie

Sydney Malama, John Bwalya Muma, Jacques Godfroid

Résumé

L'incidence de toutes les formes de tuberculose humaine (TB) en Zambie est estimée à 707/100 000. Une forte prévalence de la tuberculose bovine (BTB) - infection par *Mycobacterium bovis* - chez les bovins et les antilopes lechwe de la Kafue (*Kobus leche kafuensis*) a été signalée dans le bassin de la Kafue. La consommation de lait non pasteurisé et de produits carnés provenant d'animaux infectés représente un risque de transmission de la tuberculose zoonotique aux personnes vivant à l'interface homme-animal. Malgré la forte prévalence déclarée de la BTB chez le bétail et la faune, il n'existe pas d'informations sur la proportion de patients humains infectés par le *M. bovis* en Zambie. Ce document passe en revue les informations disponibles en anglais sur la TB de l'homme, du bétail et de la faune en Zambie, dans le but d'évaluer le fardeau des infections animales par le complexe *M. tuberculosis* et ses implications pour la santé publique.

Translated from English version into French by Jacek Sierakowski, through

TRANSLATORS
WITHOUT BORDERS

Распространение туберкулеза в Замбии в зонах контакта людей с домашним скотом и дикими животными

Sydney Malama, John Bwalya Muma, Jacques Godfroid

Краткое содержание

Расчетное распространение туберкулеза человека в Замбии составляет 707 случаев на 100 000 жителей. Отмечается, что в бассейне реки Кафуэ как среди домашнего скота, так и среди антилоп лечве (*Kobus leche Kafuensis*) широко распространен туберкулез крупного рогатого скота (возбудитель – *Mycobacterium bovis*). Употребление в пищу не подвергнутого пастеризации молока и продуктов, изготовленных из мяса зараженных животных, создает риск передачи зоонозного туберкулеза людям, проживающим в зонах контакта с домашним скотом и дикими животными. Несмотря на отмеченное широкое распространение туберкулеза крупного рогатого скота как среди домашнего скота, так и среди диких животных, информация о доле туберкулезных больных, заразившихся *M. bovis*, в Замбии отсутствует. В настоящей работе рассматриваются доступные на английском языке источники информации о распространении туберкулеза в Замбии среди людей, домашнего скота и диких животных, и делается попытка оценить бремя поражения животных комплексом *M. tuberculosis* и вытекающие из него последствия в плане охраны здоровья населения.

Translated from English version into Russian by Alexander Poddubnyy, through

TRANSLATORS WITHOUT BORDERS

Estudio sobre la tuberculosis en la interacción fauna silvestre-ganado-humano en Zambia

Sydney Malama, John Bwalya Muma, Jacques Godfroid

Resumen

La incidencia estimada de todas las formas de tuberculosis humana (TB) en Zambia es de 707/100.000. En la cuenca del río Kafue se ha hallado una alta prevalencia de tuberculosis bovina (infección con *Mycobacterium bovis,* BTB*)* en ganado vacuno y en los antílopes lechwe (*Kobus leche Kafuensis)*. El consumo de productos lácteos y cárnicos no pasteurizados provenientes de animales infectados conlleva un riesgo de transmisión de tuberculosis zoonótica a las personas que viven dentro de un área de interacción humano-animal. A pesar de la alta prevalencia de tuberculosis bovina hallada tanto en el ganado como en la fauna silvestre, no se tiene información sobre la proporción de humanos infectados con *M. bovis* en Zambia. Este documento analiza la información disponible en inglés sobre la TB en humanos, ganado y fauna silvestre en Zambia con el propósito de evaluar la carga de infecciones con el complejo *M. tuberculosis* en animales y lo que esto significa para la salud pública.

Translated from English version into Spanish by Maria Arias, through

TRANSLATORS
WITHOUT BORDERS
